# Evaluation of an e-self-management intervention (Happy Hands app) for hand osteoarthritis: Study protocol for a multicentre randomised controlled trial

**DOI:** 10.1007/s00296-025-05787-6

**Published:** 2025-01-16

**Authors:** Anne Therese Tveter, Kristine Aasness Fjeldstad, Cecilie Varsi, Marit Kristin Maarnes, Stein Jarle Pedersen, Barbara S. Christensen, Thalita Blanck, Sissel B. Nyheim, Yeliz Prior, Mathilda Björk, Tim Pelle, Ingvild Kjeken

**Affiliations:** 1 Center for Treatment of Rheumatic and Musculoskeletal Diseases (REMEDY), Health Service Research and Innovation Unit, Oslo, Norway; 2 Faculty of Health and Social Sciences, University of South-Eastern Norway, Drammen, Norway; 3 Department of Medical Services, Unit for Clinical Activity, Diakonhjemmet Hospital, Oslo, Norway; 4 Faculty of Medicine, University of Oslo, Oslo, Norway; 5 Division of Rheumatology and Research, Diakonhjemmet Hospital, Oslo, Norway; 6 Norwegian Rheumatology Association, Oslo, Norway; 7 Centre for Human Movement and Rehabilitation, School of Health and Society, University of Salford, Salford, UK; 8 Pain and Rehabilitation Center, Department of Health, Medicine and Caring Sciences, Linköping University, Linköping, Sweden; 9 Department of Primary and Community Care, Radboud University Medical Center, Nijmegen, The Netherlands

**Keywords:** Hand osteoarthritis, MHealth, EHealth, Randomized controlled trial, Smartphone app, Hand exercises

## Abstract

**Objective:**

This protocol paper describes the rationale and design of a randomised controlled trial (RCT) that aims to evaluate the (cost-)effectiveness of a 12 week e-self-management intervention (Happy Hands app) in people with hand osteoarthritis (HOA).

**Methods:**

In this multicentre RCT, 376 people with HOA will be recruited from all four health regions in Norway. Consenting participants will be randomly allocated to either a control group receiving usual care or an intervention group receiving the Happy Hands app in addition to usual care. Primary outcome will be measured at 3-months follow-up as the proportion of participants classified as OMERACT-OARSI responders (a composite score comprising change in pain, function, and disease activity), analysed using logistic regression. Secondary outcomes, including pain, hand function, stiffness, quality-of-care, health-related quality-of-life, grip strength, adherence and healthcare costs will be measured at 3- and 6-months follow-up.

**Results:**

Recruitment was initiated in November 2022 with a total of 386 participants recruited by August 2023, 194 in the intervention group and 192 in the control group. Data collection was completed in February 2024.

**Discussion:**

To our knowledge, this is one of the first large-scale, multicentre RCTs assessing the (cost-)effectiveness of a self-management program delivered through a smartphone app for people with HOA. The results from this trial can enhance our understanding of the role technology can play in managing HOA.

**Trial registration:**

NCT05568875 (https://clinicaltrials.gov/study/NCT05568875, pre-registered October 3, 2022).

## Introduction

Osteoarthritis (OA) is a prominent cause of years lived with disability worldwide [1]. The hand is one of the most commonly affected sites [2] with a lifetime risk of 50% in women and 25% in men for developing hand OA (HOA) [3]. There are no disease-modifying drugs that can cure HOA. Patient education, hand exercises, and assistive devices are the core treatments, while Non-Steroid Anti-Inflammatory Drugs (NSAIDs) can be used for a limited duration to relieve symptoms [4]. Surgery should only be contemplated if other treatment modalities are ineffective [4]. Core treatment should primarily be provided in primary healthcare, however, research shows that the quality-of-care services in general is sub-optimal [5], in particular for people with HOA [6, 7].

The use of eHealth is highlighted for self-management and better exploitation of healthcare resources [8]. An observational study suggested that digital delivery of core treatment could be a viable option in people with HOA [9], however, large methodologically sound trials of effect and cost-effectiveness are lacking [10].

In 2020, development of the Happy Hands app was initiated at Diakonhjemmet Hospital, Norway [11], with an overarching goal of delivering a standalone intervention that supports and empowers people with HOA to self-manage their disease, regardless of where they reside [11]. This paper describes the rationale and design of a multicentre randomised controlled trial (RCT) to evaluate the (cost-)effectiveness of a 12-week self-management intervention delivered through Happy Hands in addition to usual care compared to usual care only, measured as the proportion of participants classified as OMERACT/OARSI responders after 12 weeks.

## Methods

### Description of the Happy Hands app

The Happy Hands app provides users with information on recommended treatments and guidance for hand exercises. Through app notifications, participants access informative videos and new exercise sessions, each demonstrating scheduled exercises. Development and feasibility details are reported elsewhere [11]. The 12-week, evidence-based program progresses through a series of exercises, including warm-up, mobility, grip strength, stability, coordination, and stretching exercises. Each week, participants receive evidence-based informative videos on treatment options and self-management strategies, with accompanying quizzes to reinforce learning. Videos can be rewatched for continued support. To encourage adherence, participants receive weekly standardized feedback and motivational messages. They also repeatedly answer questionnaires about pain, stiffness, disease activity, and activity performance, with results visualized in graphs for self-monitoring progress.

### Design and setting

**Fig. 1:**
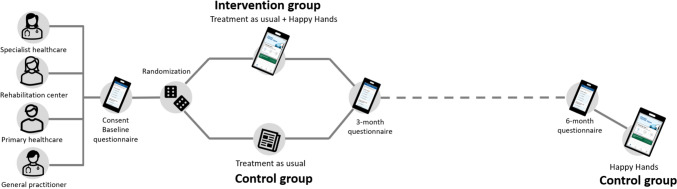
Design of the Happy Hands trial

The current project is a pragmatic multicentre parallel group superiority RCT where participants will be randomly allocated (1:1) to either a control group or an intervention group (Fig. 1). Primary endpoint will be measured at 3-months follow-up.

**Fig. 2:**
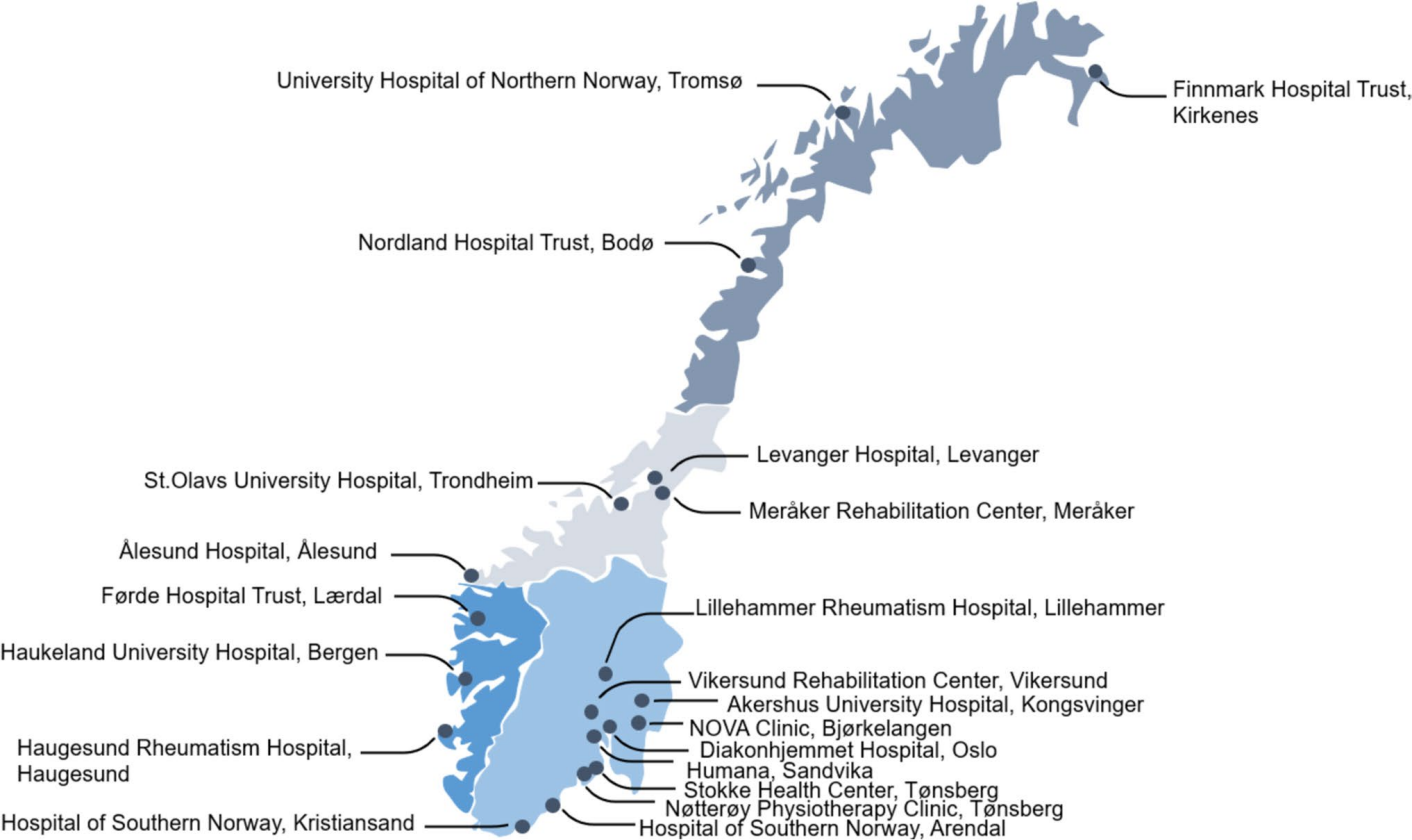
Recruiting institutions in the Happy Hands trial

Participants will be recruited by healthcare personnel from 14 hospitals, one private rheumatology centre, two rehabilitation centres, and three physiotherapy clinics from all four health regions in Norway (Fig. 2).

### Participants

People ≥ 40 years of age with symptomatic HOA diagnosed by healthcare personnel will be eligible for inclusion if they possess a smartphone and can read/understand Norwegian. They will be excluded if they have cognitive deficits, are scheduled for hand surgery within 3 months after inclusion, have serious comorbidities or inflammatory rheumatic diseases such as rheumatoid or psoriatic arthritis.

### Recruitment, randomization, and study logistics

Succeeding regular assessment by a healthcare personnel, eligible participants will be informed about the study and provided a digital consent form through Nettskjema (nettskjema.no) using electronic identification and signing through a Public Key Infrastructure solution called BankID (security level 4). After consenting, the participants will complete a digital questionnaire and will thereafter be randomly assigned to one of two distinct questionnaires (facilitated by JavaScript within Nettskjema) where they will be informed about group allocation (control or intervention), stratified according to recruitment site. Participants assigned to the intervention group will receive an URL-link to download the Happy Hands app and be provided equipment needed to perform the hand exercises shown in the app (a foam exercise ball (moderate firmness, 7 cm in diameter), tennis ball (6.5 cm in diameter), elastic band (moderate firmness, 29 cm in diameter)). Neither participants nor healthcare personnel will be blinded to group allocation.

A digital follow-up questionnaire will be sent by a project coordinator to all participants 3 and 6 months after inclusion. Recruiting institutions that are able to summon participants to follow-up consultation at 3 months will additionally assess grip strength using a JAMAR dynamometer [12] at baseline and 3 months.

### Study treatment groups

Control (usual care) group: Participants will receive usual care, which may vary from no treatment to referral to a one-day patient education program and/or occupational therapy (usually one single consultation) depending on the site. They will also be provided with a one-page information sheet containing information about HOA, common symptoms and advice on self-management.

Intervention group: In addition to usual care and the one-page information sheet, participants in the intervention group will be offered access to the Happy Hands app, guiding them through a series of information videos, exercise videos, quizzes, questionnaires, and customized feedback messages across 12 weeks [11].

Participants in both groups may seek additional treatment if necessary, including consultation with general practitioner or other healthcare personnel, prescription of NSAIDs/pain medication or a steroid injection.

### Data collection

Self-reported data will be collected for all participants at baseline, 3 and 6 months through Nettskjema. For the intervention group, additional data will be collected at baseline, after each exercise session and monthly through the app. Data accumulated through Nettskjema will be encrypted and sent to Services for sensitive data (TSD, University of Oslo), a platform for collecting and storing sensitive data. Data can be accessed through two-factor login and will only be accessible to the project manager, the PhD-candidate and approved project members.

### Outcome measures

The primary outcome measure will be the proportion of participants classified as responders according to OMERACT-OARSI responder criteria after 3 months [13]. Primary and secondary outcomes, health costs, data on use of the Happy Hands app and baseline characteristics are listed in Table 1.

**Table 1:** Description of primary and secondary outcomes, costs, app use and baseline characteristics

	Data collection instrument and scale	Timepoints
Primary outcome		
Proportion of participants classified as OMERACT/OARSI responders	The responder classification is a composite index reported as a single variable (yes/no), based on one of the two following criteria:	t0, mo, t3, t6
	*High improvement on pain or function*	
	a) ≥ 50% improvement + absolute change of ≥ 2 points in pain (NRS 0–10, 0 = no pain), OR	
	b) ≥ 50% improvement + absolute change of ≥ 0.6 point in hand function (MAP-Hand 1–4, 1 = no disability)	
	*Improvement in at least two of the three following*	
	a) ≥ 20% improvement + absolute change of ≥ 1 points in pain (NRS 0–10, 0 = no pain)	
	b) ≥ 20% improvement + absolute change of ≥ 0.3 point in hand function (MAP-Hand 1–4, 1 = no disability)	
	c) ≥ 20% improvement + absolute change of ≥ 1 points in disease activity (NRS 0–10, 0 = no disease activity)	
Secondary outcomes		
Hand pain at rest	Pain left/right hand last week, NRS 0–10, 0 = no pain	t0, ex, mo, t3, t6
Pain in activity	Pain left/right hand last week, NRS 0–10, 0 = no pain	t0, ex, mo, t3, t6
Stiffness	Stiffness left/right hand last week, NRS 0–10, 0 = no stiffness	t0, ex, mo, t3, t6
Grip strength	Maximum grip strength, mean of two measures left/right hand, in kg using the JAMAR dynamometer	t0, t3
Activity performance of the hands	Measured by the Measure of Activity Performance of the Hand (MAP-Hand); mean of 18 standardized activities, rating scale 1 ("no difficulty") to 4 ("cannot perform")	t0, mo, t3, t6
Health-related quality of life	Measured by EuroQol EQ-5D-5L, five items (mobility, self-care, usual activities, pain/discomfort, anxiety/depression) scored at a five level scare ("no problems" to "unable to do"), calculated as an utility index 0–1, 1 = best health-related quality of life. Visual analogue scale (EQ VAS), 0–100, 100 = perfect health	t0, t3, t6
Global assessment of disease activity	Disease activity last week, NRS 0–10, 0 = no disease activity	t0, mo, t3, t6
Global assessment of change	5-point Likert scale from "Much better" to "Much worse"	t3, t6
Quality of care	Modified version of the OsteoArthritis Quality Indicator questionnaire, 15 questions rated as yes/no/unsure or not applicable, scored as pass rate 0–100, 100 = best quality of care	t0, t3, t6
Motivation for hand exercises	NRS 0–10, 10 = highly motivated	t0, t3, t6
Hand exercises	Approximately number of exercise sessions each week for the last 3 months	t3, t6
Costs		
Healthcare use	Number of consultations with healthcare providers last 3 months	t3, t6
Medication use	Type and dosage last 3 months	t0, t3, t6
Hand surgery	Conducted hand surgery last 3 months (yes/no), if yes, reported sick leave due to hand surgery	t3, t6
Hospitalization	Number of hospital admissions	t3, t6
Medical equipment	Any medical equipment (i.e. hand orthoses, exercise equipment) bought last 3 months (type and cost)	t3, t6
Technical equipment	Any technical equipment (i.e. bread knife, jar key, sissors, electric toothbrush) bought last 3 months (type and cost)	t3, t6
Work status	Reported as yes/no on the following options: working, sick leave, retired, disability pension, work assessment allowance, unemployed, not working, student	t0, t3, t6
Use of the Happy Hands app (intervention group only)		
Usability	Measured by System Usability Scale (SUS), 10 statements scored on a 5-point scale from "strongly disagree" to "strongly agree"	t3, t6
Satisfaction with use of the app	NRS 0–10, 10 = highly satisfied	t3, t6
Usefulness of the app	NRS 0–10, 10 = highly useful	t3, t6
Adherence to hand exercises	Number of exercises conducted	t3, (t6)
Adherence to informational videos	Number of informational videos watched	t3, (t6)
Adherence to quizzes	Number of quizzes answered	t3
Continued use	Plans to continue using the app, measured as yes/no/unsure	t3, t6
Adverse events	Description of any complaints related to hand exercises	t3
Baseline characteristics		
Age	Years	t0
Gender	Female, male, other	t0
Living arrangement	Living alone, living together with someone	t0
Education	Elementary school, high school, university/college < 4 years, university/college ≥ 4 years	t0
Smoking	No, previously, yes	t0
Height	Cm	t0
Weight	Kg	t0
Most troublesome hand	Left, right, both	t0
Other painful joints	Hip, knee, foot/ankle	t0
Previous treatment for HOA	Yes, no	t0
Previous hand surgery	Yes, no	t0
Comorbidities	High blood pressure, heart disease, lung disease, allergy, back pain, stroke, cancer, neurological disease, diabetes, metabolic disease, mental disease, kidney disease, liver disease, ulcer, blood disease	t0
Health Literacy	eHEALS, 5-point scale with a total score ranging from 8 to 40 (higher score representing higher health literacy)	t0
Experience with using technology and digital services	Experience with smartphone, tablets, apps, video consultation, and digital services (Helsenorge.no), measured on a 5-point scale (very poorly to very good) with an additional alternative of 'never tried'	t0
Motivation for digital treatment/follow-up	NRS 0–10, 10 = to a large degree	t0

t0 = baseline; t3 = 3 months; t6 = 6 months; ex = after each exercise session (in the app/intervention group only)

*mo* Monthly (in the app/intervention group only), *NRS* Numeric rating scale, *MAP-Hand* Measure of activity performance of the hand, *HOA* Hand osteoarthritis

### Power calculations

The sample size is calculated based on the primary outcome (proportion of OMERACT-OARSI responders [13]). An OMERACT-OARSI responder rate between 26–46% is previously shown in hand exercise groups compared to 6–24% in usual care groups [14, 15]. The between-group difference in proportion of responders is estimated to be 20% [16]. With a significance level of 1%, 90% power, and between-group difference of 20%, we need to include 150 patients in each group. To account for a possible dropout-rate of 25% due to the digital nature of the study, a total of 376 participants are needed.

### Adherence

Adherence will be evaluated by tracking the number of exercise and informational videos viewed, as well as the number of quizzes completed each week. Every time a participant watches a video or submits quiz answers, this activity will be recorded in Nettskjema. Participants will also document each instance of exercise completion in the app. Recognizing that not all participants will view the same exercise videos multiple times, adherence to the exercise regimen will be based on self-reported exercise completion.

As there is no consensus regarding what constitute adequate adherence to hand exercises, the American College of Sports Medicine guidelines will be used [17]. Adherence to the exercise program will be considered adequate if participants compete ≥ 2 exercise sessions per week for a duration of least 8 weeks (67%) [18]. Adherence to information videos and quizzes will be deemed adequate if participants have watched 19 of 26 videos and completed 9 of 12 quizzes, corresponding to approximately 75% utilization.

### Adverse events (AE)

AEs will be self-reported in the 3-months follow-up questionnaire through an open-ended question and will be categorised as mild to moderate depending on the description in the questionnaire. Brief transitory pain/discomfort will be regarded as mild AEs and persistent pain/discomfort will be considered moderate AEs. Any events caused by the intervention that results in significant disability, prolonged hospitalization, life threatening events or death will be categorized as serious adverse events (SAE). It is not expected that the intervention will cause any SAEs.

### Analysis

The primary analyses will be carried out blinded to group allocation and according to the intention-to-treat principles. Primary effect analysis will be performed using logistic regression by comparing the proportion of OMERACT-OARSI responders in the two groups at 3 months, presented with odds ratio and 95% confidence interval and risk difference. Per-protocol analysis will also be performed including only those adhering to the exercise program. Secondary analyses will include between-group comparison on secondary outcomes at 3 and 6 months. Adherence and safety will be explored through descriptive statistics. Additional analyses will address predictors for treatment effect and adherence to the content of the app.

A cost-effectiveness analysis will be performed taking a healthcare perspective. The within-trial economic analysis will report on the incremental cost-effectiveness ratio (ICER) reflecting the between-group difference in incremental cost per quality-adjusted life-years (QALY). A probabilistic sensitivity analysis with 1000 replications will be calculated using bootstrap resampling method.

A statistical analysis plan (SAP) will be published in ClinicalTrials.gov prior to initiating primary analyses. Analyses will be performed using the most updated version of Stata.

### User involvement

Two patient research partners (PRPs), from Diakonhjemmet hospital and the Norwegian Rheumatism Association, have been actively engaged throughout the entire app development process [11]. They have also provided advice on recruitment strategy and feedback on the content of the information sheet and the consent form. They will continue their engagement throughout all phases of the study by discussing results and contributing with advice on how to present and disseminate results.

***Ethical considerations and dissemination.***

This study will be performed according to the Helsinki Declaration. The trial is pre-registered in ClinicalTrials.gov (NCT05568875, https://clinicaltrials.gov/study/NCT05568875) and is approved by the Regional Committees for Health Research Ethics (477,746) and the Data Protection Officer and local research committee at Diakonhjemmet Hospital. Informed consents will be signed digitally before the participants are included in the project. Participants will be informed that they can withdraw from the trial at any time without explanation. Data already included in analyses will not be deleted.

No participants, regardless of group allocation, will receive treatment that falls short of standard care. Upon completion of the study, participants in the control group will be provided with an URL-link that enable them to download the app, and necessary exercise equipment will be sent by post.

Dissemination of results will conform to Consolidated Standards of Reporting Trial (CONSORT) guidelines on Randomized Trials of Nonpharmacological Treatments [19, 20]. Results will be presented in international peer-reviewed, open access journal as well as through other relevant communication platforms.

### Timeline

Recruitment was initiated the 29th of November 2022 and was finished by the end of August 2023. A total of 386 participants were recruited, 194 were allocated to the intervention group and 192 to the control group. The final data collection (6 months follow-up) was completed by the end of February 2024. We will start analysing the results after the SAP is published by the end of 2024.

## Discussion

Although other initiatives providing digital information on core treatment to people with HOA have been developed and tested [9, 21], this is one of the first large-scale, multicentre randomised controlled trial evaluating the effect and cost-effectiveness of such an initiative in people with HOA.

Even though most participants will be recruited from a hospital setting, the trial is conducted across different healthcare levels, also recruiting participants from rehabilitation institutions and primary care, which is considered a strength for the generalizability of the results.

A possible limitation is the lack of blinding of participants and healthcare personnel, which is difficult in non-pharmacological trials. The primary outcome relies on digital questionnaires sent directly to the participants by a project coordinator, thus, the potential for bias introduced by healthcare personnel is considered minimal. Additionally, the researcher conducting the primary effect analysis will be blinded to group allocation.

Due to logistical constraints, follow-up consultations may not be arranged for all participants, resulting in some missing grip strength measurements. However, this does not affect the primary outcome, as questionnaires will be administered independently of the 3-month follow-up consultation.

We expect that the results will have an impact on the management of people with HOA, which is a large and undertreated patient group. The e-self-management intervention is expected to contribute to reduce variability and ensure health equity in treatment across healthcare levels by giving people with HOA access to information on recommended treatment and hand exercises regardless of where they reside. The results will be used in the process of implementing the Happy Hands app as part of a future HOA model of care.

## Data Availability

The dataset generated during this study is not publicly available due to GDPR regulations/local regulations at Diakonhjemmet Hospital.
